# Evaluating the impact of pass/fail United States Medical Licensing Examination Step 1 scoring on pathology residency selection

**DOI:** 10.1016/j.acpath.2023.100083

**Published:** 2023-04-25

**Authors:** Ayaka Fujihashi, Lydia C. Yang, William Haynes, Om U. Patel, Kaitlin Burge, Ishant Yadav, Nicholas Van Wagoner, Brandi McCleskey

**Affiliations:** a Marnix E. Heersink School of Medicine, University of Alabama at Birmingham, Birmingham, AL, USA; b Division of Infectious Diseases, Department of Medicine, University of Alabama at Birmingham, Birmingham, AL, USA; c Division of Forensic Pathology, Department of Pathology, University of Alabama at Birmingham, Birmingham, AL, USA

**Keywords:** Pathology residency, Pass/fail, Residency application, USMLE Step 1

## Abstract

Beginning 26 January 2022, the United States Medical Licensing Examination Step 1 changed from a numerical score to Pass/Fail. Historically, residency programs have used Step 1 scores as a valuable metric in assessing the competitiveness of applicants. We assessed how residency program criteria will change when evaluating applicants after Step 1 becomes Pass/Fail. A survey was distributed to the program directors of all 144 pathology residency programs accredited by Accreditation Council for Graduate Medical Education. Survey questions evaluated the importance of using Step 1 and Step 2 Clinical Knowledge (CK) scores when assessing applicants. Participants were asked to rank a list of applicant criteria used before and after Step 1 becomes Pass/Fail. Data were analyzed using chi-squared and paired *t*-tests with significance at *P* < 0.05. A total of 34 residency program directors (23.6%) responded to the survey. 76.5% (*P*< 0.001) of responders believed Step 1 scores were able to predict a resident's ability to pass their board exams, while 41.2% believed Step 2 CK could predict a resident's ability to pass board exams and perform clinically in pathology (*P* = 0.282). 61.8% of responders agreed that an applicant's medical school ranking would become more important (*P* = 0.001). There were no significant differences in the relative importance of 16 selection criteria after the change of Step 1 to Pass/Fail. It does not appear that Step 2 CK will become more important. Although results are constrained by a 23.6% response rate, it can be a start to guiding future students through residency applications.

The United States Medical Licensing Examination (USMLE) Step 1 is the first of three exams for medical licensure. Since 1999, students have received a three-digit score from 1 to 300, with most examinees receiving scores between 140 and 260. Though the test is designed to assess basic content knowledge for the “safe and competent practice of medicine,” it has historically been used by residency program directors as a primary screening tool for selecting candidates to interview.^1^^,^^2^ The National Resident Matching Program (NRMP) Program Director Survey from 2020 found that 90% of respondents use the USMLE Step 1 score in selecting applicants, and Step 1 score was the most cited factor used by program directors.^3^ Specifically in the field of pathology, 92% of program directors responded that they considered USMLE step 1 scores in deciding who to interview.

For 2022, the average USMLE Step 1 score among U.S. MD (allopathic) seniors that matched into pathology was 233, and the average USMLE Step 2 Clinical Knowledge (CK) score was 245. For graduates of osteopathic schools (U.S. DO seniors), the average Step 1 score was 224 and the average Step 2 CK score was 238. For U.S. citizens who graduated from international medical schools (and therefore included in the international medical graduates (IMG) cohort), these scores were 219 and 228, respectively; those who were considered non-U.S. IMGs scored 232 on Step 1 and 238 on Step 2 CK on average. By contrast, the average Step 1 score for U.S. Seniors matching into pathology in 2005 was 222. This trend of ever-increasing Step 1 score averages is certainly not unique to the field of pathology. In fact, overall average Step 1 scores have increased from 200 in 1994 to 230 in 2018. This heavy emphasis on Step 1 scores in determining an applicant's competitiveness for residency has had several unintended consequences. First, the emphasis on Step 1 has driven students to disengage from their institutional curricula in favor of third-party resources that teach to the exam. “Low yield” topics not heavily tested or on the exam at all—such as professionalism, interpersonal skills, social determinants of health, and communication—are often disregarded by students for more “high yield” topics that are largely basic science concepts.^4^ Many students begin studying prior to their dedicated Step 1 study period, further exacerbating the “parallel curriculum” of self-directed Step 1 studying and institutional preclinical lectures.^5^ Additionally, the test has significant negative impacts on students' well-being. The test has been known to induce severe anxiety among students preparing for the exam, with some students spending up to 11 hours a day during their dedicated study periods. The extreme pressure to perform well on the exam resulted in many students having their choice of specialty, career progression, or even self-worth dictated by their Step 1 score.^4^ Other critics note how Step 1 scores disproportionately affected Black and Latino students, or groups underrepresented in medicine, as these groups had significantly lower scores than their White counterparts. This manifested as Black and Hispanic applicants being three to six times less likely to receive an interview invite.^6^

In response to such criticisms, the USMLE announced on 15 September 2021 that Step 1 would transition from a three-digit score to reporting Pass/Fail for all exams taken after 26 January 2022.^7^ While this decision was made following extensive discussions with members of the medical community, others did not concur with this decision. One author argues the change to P/F scoring on Step 1 may potentially reduce students’ exposure to pathology, as the heavy emphasis of basic science concepts on Step 1 essentially mandated a basic understanding of fundamental pathologic concepts.^8^ Though this change was made with student well-being in mind, the anxiety surrounding Step 1 may be shifted to preparing and taking Step 2, which remains a numerically scored exam.

The full implications of the change to pass/fail Step 1 scoring remains to be seen. To gain clarity on how this may change the perspectives of residency program directors, we conducted a voluntary, electronic survey of program directors in the field of pathology.

Prior to the study, institutional IRB approval was obtained. The current study is part of a parent study in which the perceptions of allopathic residency program directors of 25 different specialties were examined.^9^ A fourteen-item survey was developed using Qualtrics™. Survey questions sought to elicit respondent demographics, the importance of using USMLE Step 1 and 2 CK scores when assessing applicants, and the rankings of sixteen different applicant metrics before and after the transition to pass/fail Step 1. These metrics included USMLE Step 1 score; mean number of research experiences in specialty; mean number of abstracts, posters, and presentations; Gold Humanism Honor Society membership; volunteer experiences; Alpha Omega Alpha membership; clerkship grades; Dean's letter; personal statement; preclinical grades; letters of recommendation in the specialty; class rank/quartile; away rotations in the specialty at another institution; graduate degrees; leadership and involvement; USMLE Step 2 CK score; graduation from a top 40 National Institutes of Health (NIH) funded medical school.

A link to this survey was distributed to the residency program directors and coordinators of all 144 pathology residency programs accredited by Accreditation Council for Graduate Medical Education (ACGME) using email addresses obtained through publicly available information. Responses were collected through September 2021. The data were analyzed using chi squared and paired *t*-tests with significance at *P*< 0.05. The full survey can be found in the supplementary material (Supplemental Material S1).

Thirty-four residency program directors (23.6%) responded to the survey. These respondents represented thirty programs associated with an academic hospital and four with an academic-affiliated hospital; no respondents represented community-based programs (note, there are very few of these programs accredited by the ACGME).

Notably, 76.5% of responders believed Step 1 scores were able to predict a resident's ability to pass their board exams (*P*< 0.001), but only 41.2% thought Step 2CK would be a good predictor (*P* = 0.282). None of the programs believed students would become better prepared clinically after the transition to pass/fail (*P*< 0.001). 61.8% believed a student's medical school ranking would become more important in their assessment of applications (*P* = 0.001), and 61.8% thought National Board of Medical Examiners (NBME) shelf exam scores should be shared with residency programs (*P* = 0.002) (Fig. 1).

**Fig. 1:**
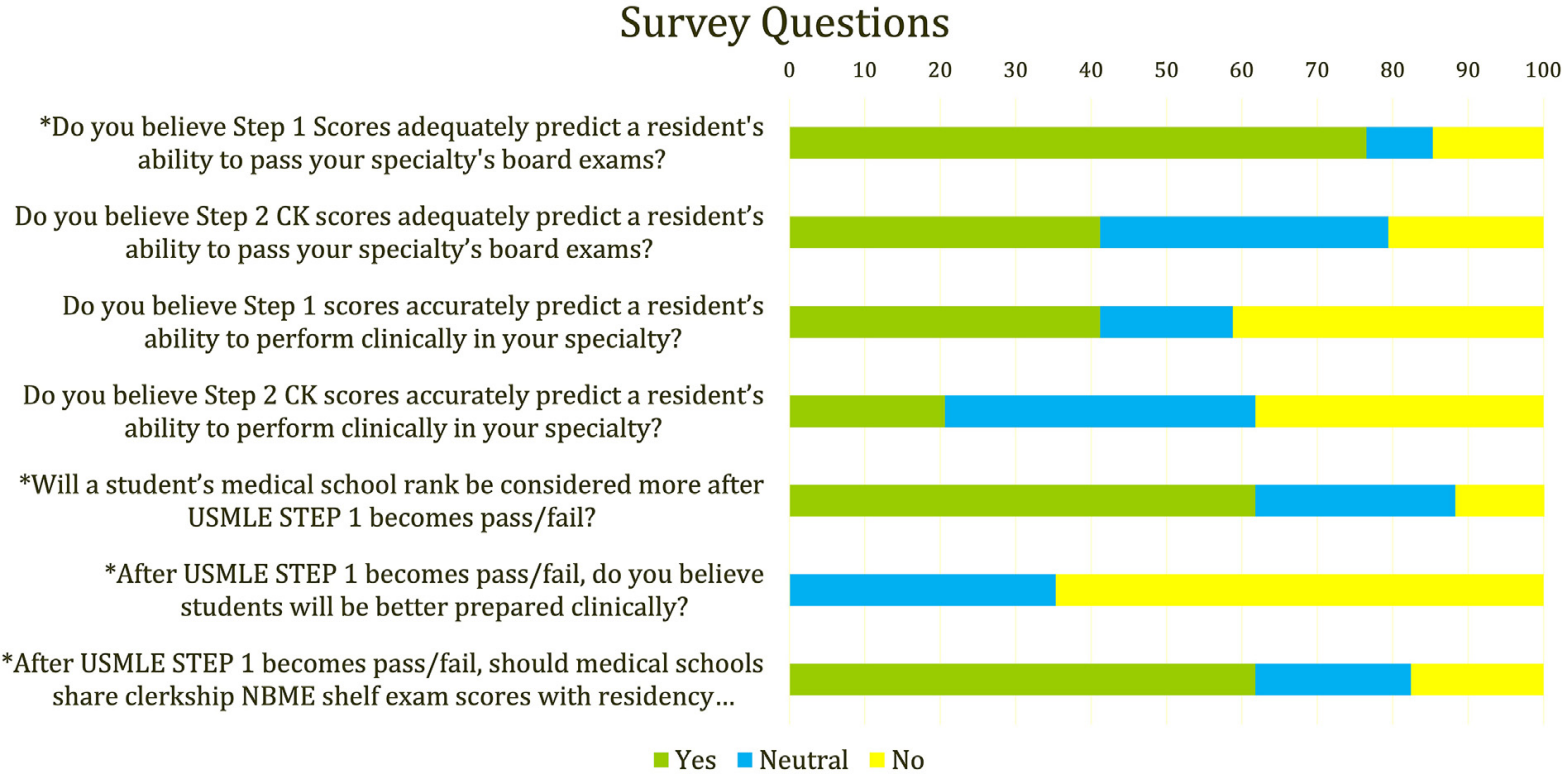
Questions asked on survey sent to pathology residency programs. Questions with significant p-values (p < 0.05) are marked with an asterisk.

There were no statistically significant changes in the relative importance of selection criteria before and after pass/fail. The top three criteria (excluding Step 1) both before and after were letters of recommendation, clerkship grades, and class rank/quartiles. Volunteer experience, being from a Top 40 NIH funded institution, and having a graduate degree remained the three least important factors in residency selection criteria (Table 1).

**Table 1:** Selection criteria ranking used before (left) and after (right) Step 1 became P/F. Changes in rankings are marked with an asterisk (∗). These are the raw rankings. There was no significant change in the relative importance of any of the 16 categories.

Before Step 1 P/F	After Step 1 P/F
Letters of Recommendation	Letters of Recommendation
Clerkship Grades	Clerkship Grades
Class Rank/Quartile	Class Rank/Quartile
Dean's Letter	Dean's Letter
Step 2 CK	Pre-Clinical Grades∗
Personal Statement	Step 2 CK∗
Pre-Clinical Grades	Personal Statement∗
Mean Number of Research Experiences in Specialty	Mean Number of Research Experiences in Specialty
Number of Abstracts and Publications	Number of Abstracts and Publications
Away Rotation in Pathology	AOA
AOA	Away Rotation in Pathology
Involvement and Leadership	GHHS
GHHS	Involvement and Leadership
Volunteer Experience	Volunteer Experience
From a Top 40 NIH Funded Medical School	From a Top 40 NIH Funded Medical School
Graduate Degree	Graduate Degree

This study is the first to our knowledge to examine changes in selection criteria for pathology residency program directors specifically. Overall, the results show that Step 2 CK will not become more important after Step 1 transitions to P/F. Rather, residency programs do not believe P/F will better prepare students to pass board exams or perform clinically. The relative importance of selection criteria also remains the same. In contrast, studies looking at other specialties, such as diagnostic radiology and interventional radiology, found that Step 2CK would become an important part of a student's application.^10^

Pathology is a specialty that medical students have minimal exposure to after the preclinical years. The subject makes up 44–52% of Step 1 per USMLE content guidelines and is highly emphasized during the first two years of medical school.^11^ Thus, it is not surprising that residency programs view Step 1 as a more important metric in assessing applicants than its clinically focused counterpart, Step 2 CK. After P/F, class rank will become an important factor in a resident's application. However, class rank is highly dependent on medical schools' grading policies, which vary between institutions. Additionally, some medical schools opt not to provide information on a students' rank on their Medical Student Performance Evaluation, a summary letter of evaluation submitted to residency programs. The only other nationally standardized metrics are Step 2 CK, NBME shelf exam scores, and the Comprehensive Osteopathic Medical Licensing Examination (COMLEX), the national board exam for osteopathic students.

In addition, COMLEX Level 1 remained a three-digit score until 10 May 2022.^12^ Approximately one in every five medical students is attending an osteopathic medical school and thus will be taking COMLEX board examinations.^13^ The recent growth of osteopathic medical schools is reflected in the field of pathology, as the percentage of pathology residency spots filled by osteopathic medical students has increased from 6.6% in 2011 to 12.0% in 2022.^13^ With both Step 1 and COMLEX Level 1 becoming P/F but at different times, it is likely that more osteopathic students will be applying with a three-digit score until the NRMP Match Cycle of 2024. This may make it more difficult to fairly compare applicants between allopathic and osteopathic schools for the next few application cycles.

Overall, the transition to P/F takes away a key objective metric traditionally used by pathology residencies. Our results showing that Step 2 CK will not play a larger factor in assessing applicant success can offer some relief to medical students interested in pathology, as this exam is typically taken at the end of a student's third year of medical school and only a few months away from Electronic Residency Application Service submissions. If residency programs were to place the same weight to Step 2 CK scores as they did for Step 1, medical students would not be able to gauge their competitiveness until the end of their third year. Our survey results suggest that students interested in pathology should continue to secure strong letters of recommendations and perform strongly in clerkships, the same factors that were important before the transition to P/F. Seeking additional exposure to pathology to obtain letters of recommendation from Pathologists is also strongly encouraged. Though our results showed that there was no statistically significant change in selection criteria ranking, this may change with successive application cycles as residency selection committees learn to assess applicants without Step 1 scores.

Our study also revealed that residency program directors believe Step 1 scores can help predict how applicants will perform on certification examinations administered by the American Board of Pathology. Furthermore, none of the program directors believed the change to P/F reporting would improve students’ clinical preparedness. This raises potential concerns over the downstream effects of Step 1 on residency performance and board examination pass rates. While the stress and anxiety surrounding Step 1 preparation has decreased, it is important to consider whether the burden has been alleviated or simply delayed to Step 2 CK or even residency training.

Whether the transition to a P/F Step 1 will achieve its aims remains to be seen. The implementation of P/F aims to promote a culture of fairness and equity by assessing residency applicants holistically through several factors. There is concern that emphasizing criteria such as medical school ranking or research involvement may put students from less prestigious medical schools or those with fewer access to research opportunities at a disadvantage.^14^ However, the removal of Step 1 scoring may also highlight applicant characteristics through clinical skills and letters of recommendations. The pressure to perform exceptionally on a single exam—and the consequently associated stress and burnout—is alleviated and distributed across a more holistic application. Residency committees will continue to re-evaluate their selection criteria with every application cycle, and the results of this survey only reflect program director perspectives at one point in time. As residency selection committees gain experience in evaluating applicants though utilization of a multi-factorial review process, the transition to pass/fail Step 1 has potential to lead to a more diverse and well-rounded physician workforce.

One main limitation to our study is a survey response rate of 23.6%, and we acknowledge it is difficult to draw conclusions from a limited sample size. In addition, the survey was initially sent out in 2021, and it is possible that programs have formed new opinions on selection criteria as we approach the first application cycle to have primarily P/F Step 1 scores. Future studies examining the correlation between Step 1/Step 2CK scores and pathology board pass rates are warranted to determine which exam is a better predictor of residency and board exam preparedness.

USMLE Step 1 has historically been used by pathology residency programs as an important criterion in assessing applicants. The transition from a three-digit to a binary P/F score did not change the relative importance of selection criteria, and our survey results show that Step 2CK will not increase in importance. The variability in medical school curriculum and grading as well as the transition to COMLEX Level 1 to P/F may make it difficult to fairly compare applicants during future application cycles.

## Funding

The article processing fee for this article was funded by an Open Access Award given by the Society of ‘67, which supports the mission of the Association of Pathology Chairs to produce the next generation of outstanding investigators and educational scholars in the field of pathology. This award helps to promote the publication of high-quality original scholarship in *Academic Pathology* by authors at an early stage of academic development.

## Declaration of competing interest

The Authors declare that there are no competing interests.
