# Supplementary material for: Evaluating the impact of pass/fail United States Medical Licensing Examination Step 1 scoring on pathology residency selection
Source: Acad Pathol. 2023 Apr 25;10(2):100083. doi: 10.1016/j.acpath.2023.100083 (PMC10164826; doi:10.1016/j.acpath.2023.100083)
Supplement: Multimedia component 1 [file mmc1.docx]

**Supplemental Material S1**

Survey Questions

Q0 Please indicate your position from the choices below.

- Program Director
- Associate Program Director
- Assistant Program Director

Q1 Please choose your specialty from the dropdown menu below.

- Internal Medicine
- Urology
- Neurosurgery
- Orthopedics
- Otolaryngology
- Dermatology
- Vascular Surgery (Integrated)
- Cardiothoracic Surgery (Integrated)
- Interventional Radiology
- Radiation Oncology
- Obstetrics and Gynecology
- Neurology
- Ophthalmology
- General Surgery
- Plastic Surgery (Integrated)
- Diagnostic Radiology
- Emergency Medicine
- Family Medicine
- Pathology
- Pediatrics
- Physical Medicine and Rehabilitation
- Psychiatry
- Internal Medicine/Pediatrics
- Child Neurology
- Anesthesiology

Q2 Are you a top 15 NIH funded program?

- Yes
- No
- Don't Know

Q3 How many applications do you receive per year (estimate)?

________________________________________________________________

Q4 How many open spots do you have per year?

________________________________________________________________

Q5 Are you an academic, academically-affiliated, or a community-based program?

- Academic
- Academic-affiliated
- Community-Based

Q6 After *USMLE STEP 1* becomes pass/fail, should medical schools share clerkship NBME shelf exam scores with residency programs?

- Yes
- Neutral
- No

Q7 Do you believe that *USMLE STEP 1* scores adequately predict a resident's ability to pass your specialty's board exams?

- Yes
- Neutral
- No

Q8 Do you believe that *USMLE STEP 2CK* scores adequately predict a resident's ability to pass your specialty's board exams?

- Yes
- Neutral
- No

Q9 Do you believe that *USMLE STEP 1* scores accurately predict a resident's ability to perform clinically in your specialty?

- Yes
- Neutral
- No

 Q10 Do you believe that *USMLE STEP 2CK* scores accurately predict a resident's ability to perform clinically in your specialty?

- Yes
- Neutral
- No

Q11 Will a student's medical school rank be considered more after *USMLE STEP 1* becomes pass/fail?

- Yes
- Neutral
- No

Q12 After *USMLE STEP 1* becomes pass/fail, do you believe students will be better prepared clinically?

- Yes
- Neutral
- No

Q13 Please rank the following factors for a residency application, with 1 being the factor of most importance (***prior*** to STEP 1 going P/F)

______ USMLE STEP 1 Score

______ Mean number of Research Experiences in Specialty

______ Number of Abstracts, Presentations, and Publications

______ Gold Humanism Honor Society (GHHS) membership

______ Volunteer Experience

______ Alpha Omega Alpha (AOA) membership

______ Clerkship Grades

______ Dean's Letter

______ Personal Statement

______ Preclinical Grades

______ Letters of Recommendation in the Specialty

______ Class Rank/Quartile

______ Away rotation in your specialty at another institution

______ Applicant has Graduate Degree (PhD, MPH, MBA etc.)

______ Involvement and Leadership

______ USMLE STEP 2 CK Score

______ Graduated from one of the 40 U.S. medical schools with the highest NIH funding

Q14 Please rank the following factors for a residency application, with 1 being the factor of most importance (***after*** STEP 1 going P/F)

______ Mean number of Research Experiences in Specialty

______ Number of Abstracts, Presentations, and Publications

______ Gold Humanism Honor Society (GHHS) Member

______ Volunteer Experience

______ Alpha Omega Alpha (AOA) Member

______ Clerkship Grades

______ Dean's Letter

______ Personal Statement

______ Preclinical Grades

______ Letters of Recommendation in the Specialty

______ Class Rank/Quartile

______ Away rotation in your specialty at another institution

______ Applicant has Graduate Degree (PhD, MPH, MBA etc.)

______ Involvement and Leadership

______ USMLE STEP 2 CK Score

______ Graduated from one of the 40 U.S. medical schools with the highest NIH funding
